# Innovative Tools for Mechanobiology: Unraveling Outside-In and Inside-Out Mechanotransduction

**DOI:** 10.3389/fbioe.2019.00162

**Published:** 2019-07-16

**Authors:** Danahe Mohammed, Marie Versaevel, Céline Bruyère, Laura Alaimo, Marine Luciano, Eléonore Vercruysse, Anthony Procès, Sylvain Gabriele

**Affiliations:** ^1^Mechanobiology and Soft Matter Group, Interfaces and Complex Fluids Laboratory, Research Institute for Biosciences, University of Mons, Mons, Belgium; ^2^Department of Neurosciences, Research Institute for Biosciences, University of Mons, Mons, Belgium

**Keywords:** mechanobiology, mechanotransduction, cytoskeleon, integrins, microsystem and macrosystem approaches, force, signaling/signaling pathways

## Abstract

Cells and tissues can sense and react to the modifications of the physico-chemical properties of the extracellular environment (ECM) through integrin-based adhesion sites and adapt their physiological response in a process called mechanotransduction. Due to their critical localization at the cell-ECM interface, transmembrane integrins are mediators of bidirectional signaling, playing a key role in “outside-in” and “inside-out” signal transduction. After presenting the basic conceptual fundamentals related to cell mechanobiology, we review the current state-of-the-art technologies that facilitate the understanding of mechanotransduction signaling pathways. Finally, we highlight innovative technological developments that can help to advance our understanding of the mechanisms underlying nuclear mechanotransduction.

## Introduction

During the last two decades, increasing evidence has suggested that the physico-chemical properties of the cell microenvironment and the physical forces exerted by cells and tissues play critical roles in the regulation of physiological and pathological situations. In both contexts, cells must adapt their behavior by converting physical signals into biochemical signals and changes in gene expression by using mechanochemical transduction, or mechanotransduction, signaling pathways.

**Figure 1:**
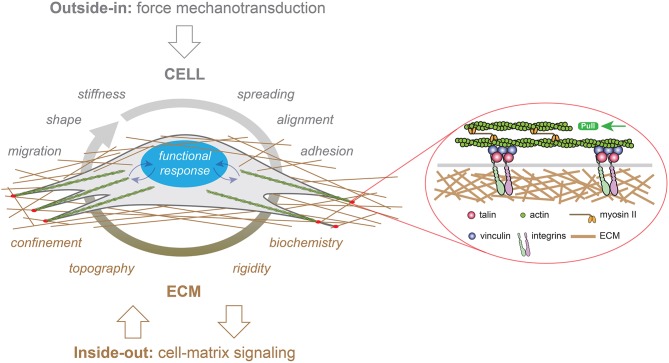
Representation of “outside-in” (in gray) and “inside-out” (in brown) mechanotransduction signals in a cell growing in a three-dimensional (3D) fibrous matrix. External forces applied to eukaryotic cells modulate their migration, shape, stiffness, spreading, alignment or adhesion behaviors, whereas the ECM provides multiple cues to cells such as confinement, topography, rigidity and biochemistry. Focal adhesion (FAs, in red) serve as crucial sites for both outside-in and inside-out mechanotransduction through the recruitment of transmembrane integrins. Mechanical signals (curved blue arrows) are converted in biological responses by the nucleus, which is depicted in blue.

Adherent cells are connected to the extracellular matrix (ECM) through the transmembrane receptor integrins. Mechanical signals can be detected via focal adhesion (FA) sites and translated into biochemical information through integrin-related signaling pathways. The inside tension generated by the actomyosin contractility can be transferred to the ECM through integrins at FAs. As a consequence, FAs serve as crucial sites for both outside-in and inside-out mechanotransduction (Figure 1). Although it is clear that integrins play a crucial role in translating outside-in and inside-out signals, it remains unclear how cells can be able to sense mechanical forces and convert mechanical signals into biological responses (Jaalouk and Lammerding, 2009). Moreover, this global mechanism is further complicated by the highly dynamic behavior of cells that can adapt their morphology and cytoskeletal organization in response to mechanical forces. In addition, specialized mechanoreceptors can enhance mechanosensation for critical processes such as blood pressure, auditory function or touch sensation by using specific organelles that can detect a wide range of stimulus frequencies (Peng et al., 2011). For instance, the so-called “hair” cells transduce mechanical vibrations into electrical signals that propagate to the brain. Located on the apical surface of sensory hair cells, hair bundles are filled with actin, named stereocilia. The mechanical deflection of the stereocilia toward the tall edge opens gated ion channels, while opposite deflection closes channels (Katta et al., 2015). Indeed, the actin filament sliding generates a force that changes the conformation of a transmembrane protein, leading to the transient entry of calcium ions. Other mechanisms such as the modifications of intracellular protein conformations or the direct transmission of forces to the cell nucleus are only being explored (Chu et al., 2019). To address this issue, the mechanobiology field has become very active at the forefront of current research (Roca-Cusachs et al., 2017). Mechanobiology is an interdisciplinary field that focuses on physical forces and their impact on cell mechanics. A major challenge in mechanobiology is to understand mechanotransduction mechanisms by which mechanical signals are transduced into a cascade of biochemical events (Humphrey et al., 2014) and to understand how these molecular events contribute to development, physiology and disease.

To answer these open questions, current studies need to stimulate mechanically living cells and tissues and then determine their mechanical and functional responses. Current studies on cell mechanotransduction are mostly limited by the techniques that are available to impose mechanical stimulation at both single cell and tissue levels and, in turn, allow to measure biomechanical and biochemical cell responses. Here, we introduce some basic concepts of mechanobiology and we review recent experimental developments that have significant implications for addressing challenging questions in cellular mechanotransduction.

## Basic Concepts of Mechanobiology

The concept of force in cell biology can be intuitively related to pushing or pulling actions exerted by individual or assembly of cells. Even if the concept of force in cell biology is difficult to define, it exists a wide range of physiological situations where mechanical events are crucial to the establishment of cellular functions. For instance, the division of eukaryote cells requires the assembly of a bipolar spindle, which the morphology mostly depends on the activities of molecular motors that generate pushing or pulling forces on the microtubule-based mitotic spindle (Shimamoto et al., 2015). The quantification of cellular forces requires the development of micro- or nano-sensors capable of converting forces into mechanical deformations, knowing the material properties of the force sensor (i.e., the spring constant or the elastic modulus). Micro- or nano-sensors provide therefore an exact readout of forces from the material deformation within a range from few piconewtons to hundreds of nanonewtons.

**Figure 2:**
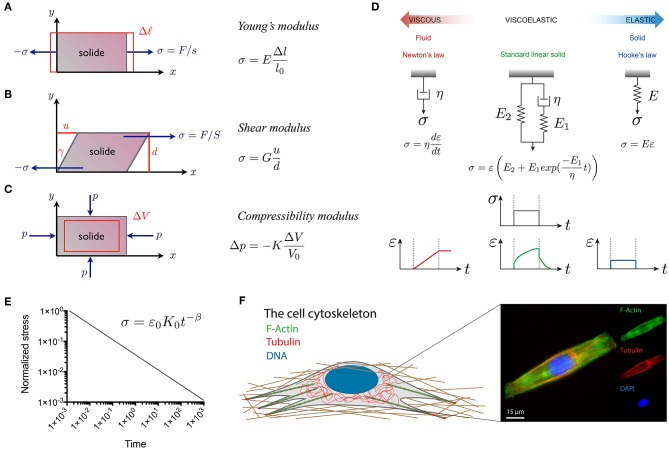
Representation of the fundamental quantities involved in the three main modes of deformation used in material characterization: **(A)** linear elongation, **(B)** shear deformation and **(C)** isotropic compression. **(D)** Viscoelastic models, such as the standard linear model in green, can be obtained from the association of dashpots, that represent viscous Newtonian fluids (in red), and springs that represent ideal elastic solids (in blue). **(E)** The power law type of relaxation in log–log scale is a line with a slope β, which is the power law exponent. The power law exponents are β = 0 and β = 1 for purely elastic and viscous materials, respectively. Elastic and viscous mechanisms contribute to a complex relaxation response when 0 < β < 1 (Grevesse et al., 2015). **(F)** The composition and spatial organization of cytoskeletal components significantly influence the cell rheological properties. Epifluorescence image of a C2C12 myoblast immunostained for F-actin (in green), microtubules (in red) and DNA (in blue). The scale bar is 15 μm.

Force assays allow to probe the mechanical properties of cells, which describe the cell deformation over time in response to an applied stress. The elastic modulus corresponds to the scaling between stress and strain of cells and depends on the deformation mode (Figures 2A–C). The Young's modulus corresponds to the cell elasticity with a unit of pascals (Pa) under extension. This fundamental property of living cells and tissues is involved in the establishment of their three-dimensional (3D) shapes under a mechanical stress. However, one must consider that living cells and tissues need to be considered as viscoelastic living materials (Figure 2D). Viscoelastic materials undergoing a mechanical deformation store and dissipate mechanical energy (Coppée et al., 2011). The viscoelastic behavior of cells and tissues leads to the relaxation of the mechanical stress and the increase of the deformation over time (Moeendarbary and Harris, 2014) (Figures 2E,F). Various rheological models, such as the standard linear solid model, have been proposed to describe the viscoelastic behavior of cells and tissues (Figure 2D).

In addition to the investigation of the mechanical properties of cells themselves, forces applied by cells on their surrounding are key for understanding inside-out mechanotransduction pathways. Contractile cellular forces are transmitted to other cellular neighbors via cell-cell adhesive interactions (i.e., cadherins) and to their local microenvironment through cell-matrix interactions (i.e., integrins). Cellular tractions forces occur across small-length scales (nano- to micrometers) in the range of pico to nanonewtons, making challenging a direct experimental measurement. Interestingly, ion channels have been proposed for decades to be central for sensing mechanical forces, but their identity remained largely elusive until the discovery of Piezo 1 and Piezo 2 channels (Coste et al., 2010). The current view suggests that cellular mechanotransduction signaling is largely mediated by transmembrane proteins, however the Piezo1 ion channel has been suggested to be involved in the emergence of traction forces and can therefore revisit the current concept (Murthy et al., 2017; Nourse and Pathak, 2017).

In the human body, the magnitude of forces exerted by cells varies significantly and mainly depends on the physiological location of the cells. Indeed, each type of tissue construct is characterized by both specific physico-chemical and mechanical properties. For this reason, a large effort has been made in the last two decades to propose novel synthetic materials that can recapitulate the physico-chemical properties of the complex native cellular microenvironment.

## Synthetic Matrices to Reproduce the Complexity of the Native Cell Microenvironment

In human tissues, the ECM is a highly 3D dynamic structure where cells have interactions with a myriad of biochemical (e.g., soluble factors) and biophysical (e.g., stiffness/stress) cues that direct their functions. Cells are constantly remodeling the ECM through synthesis, degradation and chemical modifications. These processes imply important changes in ECM properties (e.g., stiffness and porosity), which in turn drive cell fate and maintain tissue homeostasis (Baker et al., 2015). The biochemical composition of the ECM is complex and mainly consists of branched glycosaminoglycan structures (e.g., heparin sulfate and chondroitin sulfate) (Karp, 2015) and high molecular weight proteins (e.g., collagen, fibronectin, and laminin). All of these macromolecules form a 3D fibrillar network that provides a unique bioactive micro-environment. Most of the ECM components contain adhesive binding sites that are involved in the transduction of mechanical signals exerted from the local micro-environment (Humphries, 1990). The mechanisms used by cells to receive and process mechanical signals are still not understood due to the complexity of the native cell micro-environment. In addition, cell-ECM interactions typically involve coordinated presentation of multiple factors (e.g., cell-ligand density, porosity or stiffness) that can be presented over multiple time scales. To overcome this barrier, smart hydrogels have emerged as a promising alternative strategy to standard plastic dishes for cell culture.

**Figure 3:**
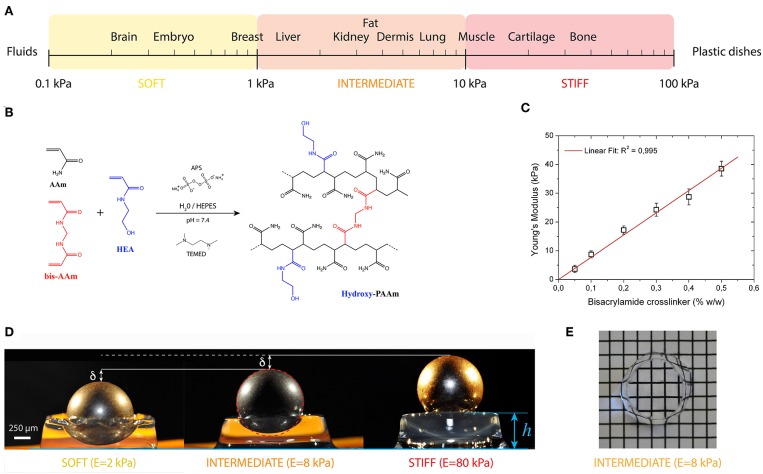
**(A)** The elasticity of living tissues spans a wide range of rigidities which are organized in three domains: soft (0.1 ≤ E ≤ 1kPa), intermediate (1 ≤ E ≤ 10 kPa) and stiff (10 ≤ E ≤ 100 kPa). **(B)** Acrylamide (AAm, in black) and bisacrylamide (bis-AAm, in blue) and N-hydroxyethylacrylamide (HEA, in red) monomers were co-polymerized to form a hydrophilic network of polyacrylamide containing hydroxyl groups (hydroxy-PAAm) by random radical polymerization (Grevesse et al., 2013, 2014). **(C)** The amount of bis-AAm cross-linker allows to modulate the stiffness of hydroxy-PAAm hydrogels. **(D)** Images of three hydroxy-PAAm hydrogels of various rigidities (from left to right: soft in yellow, intermediate in orange and stiff in red) deformed by a static steel ball that exerts a constant load. The resistance of the hydroxy-PAAm hydrogels against the deformation imposed by the steel ball is proportional to the elastic modulus of the hydrogels. **(E)** Hydroxy-PAAm hydrogels have superior optical properties, such as high transparency, that do not depend on their mechanical properties.

Hydrogels are defined as crosslinked water-swollen biomacromolecules that form a three-dimensional structure. The large amount of water in hydrogels allows the diffusion of biomolecules secreted by cells. The design of hydrogels suitable for cell culture requires to reproduce both the biochemical and mechanical properties of their native microenvironment. Water-swollen polymers such as poly(ethyleneglycol) (PEG), poly(vinyl alcohol) (PVA), poly(2-hydroxyethylmethacrylate) (PHEMA) and poly(acrylamide) (PAAm) can form elastic hydrogels that can reproduce some basic mechanical aspects of soft tissues (Figure 3A) (Annabi et al., 2014). Although still not fully understood, growing evidence suggests that cells interpret elasticity through integrin-mediated mechanotransduction signals that trigger outside-in signaling cascades. The seminal work of Engler and coworkers was performed with polyacrylamide hydrogels in order to modulate human mesenchymal stem cells (hMSCs) differentiation by tuning the hydrogel elastic modulus (Engler et al., 2006). Promising matrix candidate for studying mechanobiology *in vitro*, PAAm hydrogels have received a large attention in order to overcome its native non-adhesive properties, for instance using heterobifunctional cross-linker sulfo-SANPAH (Wang and Discher, 2007), hydrazine and protein oxidation by periodate (Damljanovic et al., 2005), deep UV exposure (Tseng et al., 2011) or HS-ester during the polymerization phase (Polio et al., 2012). However, most of these methods present difficulties for controlling easily the cell–ligand density and decoupling the relative contribution of mechanotransduction cues. In order to address this limitation, Grevesse et al. introduced a new PAAm hydrogel, called hydroxy-PAAm, that incorporates hydroxyl groups to allow the functionalization of PAAm hydrogels with ECM proteins with minimal requirements in cost or expertise (Figure 3B) (Grevesse et al., 2013). Hydroxy-PAAm has been shown to be an effective biomaterial for immobilizing any desired proteins and tuning important physico-chemical parameters of the matrix (Grevesse et al., 2014), such as ligand density and matrix stiffness (Figures 3C,D), while keeping superior optical properties (Figure 3E). For instance, hydroxy-PAAm hydrogels were used to show the individual role of αvβ3 and α5β1 integrins in the matrix rigidity sensing of highly motile cells (Riaz et al., 2016) and that matrix rigidity can modulate the axon growth, the density of synapses and the electrophysiological activity of neuronal networks (Lantoine et al., 2016). Due to the elastic nature of PAAm hydrogels, most of the cellular mechanotransduction studies have converged upon the idea that ECM elasticity is the main physical cue sensed by cells. However, *in vivo* matrices exhibit viscoelastic behavior characterized with stress relaxation properties that can regulate cell functions (Chauduri et al., 2015, 2016; Bauer et al., 2017; Vining et al., 2019). Recently, Charrier et al. reported the synthesis of gels with an independent tuning of elastic and viscous moduli. By altering systematically the hydrogel viscosity, the authors demonstrated the time dependence of cellular mechanosensing and the influence of viscous dissipation on cell phenotype (Charrier et al., 2018). Despite many advantages to mimic the structure of native tissues, one major drawback of PAAm hydrogels is that porosity changes with variations in stiffness, leading to changes in cell-fate decisions (Trappman et al., 2012).

In addition to these works, magnetic hydrogels (M-gel systems) (Niland et al., 2001) and photoresponsive hydrogels (PRHs) that include photochromic chromophores as the photoreactive groups within the 3D hydrogels network (Tomatsu et al., 2011) were developed to mimic the mechanical environment of the ECM (Dong et al., 2018). Diverse photoreactions have been used to tune the properties and functions of hydrogels such as degradability (Kloxin et al., 2009), polarity (Liu et al., 2005) or adhesion (Bryant et al., 2007), which has made photoresponsive hydrogels useful for engineering a dynamic cell microenvironment for mechanotransduction assays (Zhang et al., 2015).

Even if considerable efforts have been made to design synthetic hydrogels with finely tunable physico-chemical and mechanical properties, ECM fiber networks remain more complex than their synthetic analogs. Indeed, native ECM fibers can be mechanically stretched by cell-generated forces that will upregulate their Young's modulus (Liu et al., 2006), activate cryptic sites (Klotzsch et al., 2009) or inhibit binding sites (Chabria et al., 2010; Kubow et al., 2015). Furthermore, because most ECM fibers, such as fibronectin, have enzymatic cleavage sites, particularly for metalloproteinases (MMPs), they can be enzymatically degraded causing the release of peptide fragments that may play a crucial role in regulating inflammatory processes (Modol et al., 2014). In addition to MMP-degradable hydrogel platforms (Lueckgen et al., 2018; Xiaomeng et al., 2018), novel technologies to create synthetic matrices with stretched fibers will be essential to learn whether and how cell-cell and cell-ECM mechanotransduction crosstalk is regulated by ECM fiber tension (Vogel, 2018).

## Standardizing Cell-Substrate Interactions With Microfabricated Tools

Interactions of cells with the ECM determine their fate through the modulation of cell shape, cell-surface adhesions and cell spreading. The ability to produce precisely engineered surfaces for cell culture that can provide robust *in vitro* assays to control cell adhesion is crucial for understanding inside-out and outside-in mechanotransduction signals.

**Figure 4:**
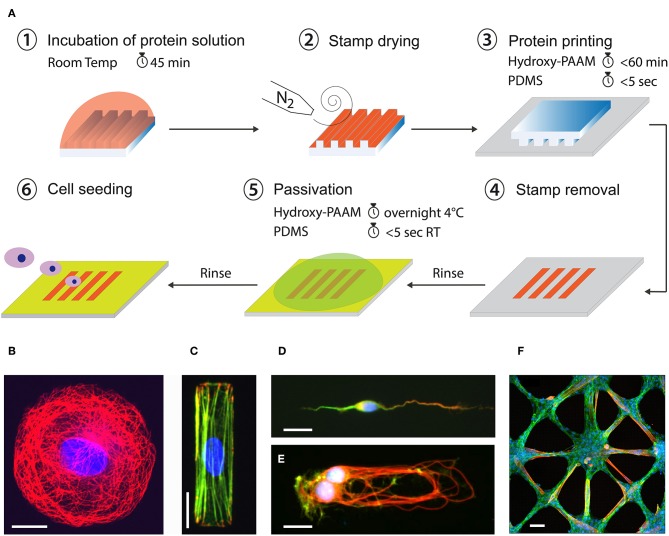
**(A)** Microcontact printing allows to from 2D micropatterns of proteins on flat culture substrates from a PDMS structured microstamp (Grevesse et al., 2013; Versaevel et al., 2014b). Example of fluorescent images of different cell types grown at the single cell and tissue levels on adhesive micropatterns of various geometries and protein coatings. Human Umbilical Vein Endothelial Cell (HUVEC) were grown on **(B)** a circular micropattern of fibronectin (FN) and stained for tubulin (in red) and DNA (in blue), **(C)** a rectangular micropattern of FN and stained for vinculin (in red), actin (in green) and DNA (in blue). **(D)** A cortical neuron was grown on a stripe of laminin (LM) and stained for MAP2 (in green), Tau (in red) and DNA (in blue). **(E)** A doublet of cortical neurons was grown on a rectangular micropattern of LM and stained for tubulin (in red), actin (in green), and DNA (in blue). **(F)** A 2D tissue of C2C12 myoblasts was grown on a star-shaped micropattern of FN and stained for actin (in green), troponin T (in red) and DNA (in blue) after differentiation in myotubes. Scale bars are **(B)** 10 μm, **(C)** 20 μm, **(D)** 10 μm, **(E)** 20 μm, and **(F)** 50 μm.

In conventional two-dimensional (2D) cultures, cells grow until confluence without any specific spatial organization. Major drawbacks of conventional cultures are therefore the difficulty to manage complex parameters involved in mechanotransduction signaling pathways. To address this limitation, a large effort has been made during the last two decades to develop robust micropatterning techniques for manipulating cell adhesion patterns. Although the first micropatterning methods were introduced more than 40 years ago (Carter, 1967; Harris, 1973), they only became commercially available recently. Among a wide range of patterning techniques, the microcontact printing (μCP) technique mainly developed by the Whitesides group at Harvard University (Whitesides et al., 2001) has become the most popular and widely used technique for cell biology assays (Figure 4A). Controlling cell adhesion through adhesive micropatterns allows to impose boundary conditions in cell culture in order to control both cell shape and structure. Cell shape can be precisely controlled to minimize variations of cell morphologies inherent to any cell types (Figures 4B–E). Adhesive micropatterns were used to demonstrate that cells need to spread, to extend spatially, to generate forces and not only to have the biochemical factors to survive and grow (Chen et al., 1997). Micropatterns have been used for instance to demonstrate that the geometry of the adhesive micropattern influences the cell division through the reorientation of the mitotic spindle (Thery et al., 2005; Fink et al., 2011). Using endothelial cells grown on a wide range of micropattern geometries, it has been shown that compressive forces exerted by the actomyosin filaments regulate nuclear orientations and deformations (Versaevel et al., 2012). Changes in cell shape imposed by the geometry of the adhesive micropattern modulate cell proliferation through chromatin condensation, demonstrating a mechanotransduction signaling pathway. It has been shown that the direction of the leading-edge extension can be controlled by constraining cell shape using adhesive micropatterns. For instance, square cells were found to reorient cell-substrate adhesions and stress fibers, concentrating therefore contractile forces in the corner regions (Parker et al., 2002). Using μCP, it has been suggested that mechanical interactions between cells and ECM that modulate cytoskeletal tension may therefore play a key role in the control of directional cell motility. In the context of cell migration, μCP was also used to study the influence of the 2D spatial confinement by using adhesive ratchets (Mahmud et al., 2009; Mohammed et al., 2019). Adhesive micropatterns were applied to the study of the influence of the adhesive micro-environment on the actin architecture and contractility (Mandal et al., 2014). The study of the influence of the geometry on multicellular systems (Figure 4F) has shown that cells propagate the alignment observed at the edges over hundreds of micrometers into the cell monolayer (Duclos et al., 2014). Wide adhesive stripes have been used to demonstrate that cells self-organize in a nematic phase developing a shear flow close to the edges, while the cells align perfectly with the direction of the stripe and the net flow vanishes on stripes narrower than a critical width (Duclos et al., 2018). More recently, micropatterns were used to study how cells distinguish between positive and negative curvatures in their physical environment. The authors found that concave edges promote polarized actin structures with actin flow directed toward the cell edge, whereas convex edges were characterized by an actin retrograde flow (Chen et al., 2019). Proteins micropatterns have also started to be used for studying early embryonic spatial patterning during development (Warmflash et al., 2015). For instance, a neuroectoderm model based on micropatterned human pluripotent stem (hPS) cells was developed by Xue et al. for mimicking *in vitro* the neuroectomderm regionalization observed during early neurulation *in vivo* (Xue et al., 2018). Most of the previous studies that controlled cell shape for studying cell-matrix interactions have been done using 2D micropatterned surfaces. However, a large number of cell types experience *in vivo* complex 3D environments with different physico-chemical properties.

The design of well-defined micro- and nano-structured surfaces can help to understand interactions of cells with topographical features. It has been observed that epithelial cells align along the preferential direction of nano-grooves, suggesting that the ECM topography encountered by cells *in vivo* is involved in cell polarization (Teixeira et al., 2003). Interestingly most of the components of the cytoskeleton were found to be correlated to the cell orientation, as well as the nucleus. By patterning microchannels using polyacrylamide hydrogels with a stiffness ranging from 400 Pa to 120 kPa, Pathak and Kumar found that migration velocity increased with the matrix stiffness in narrow microchannels (Pathak and Kumar, 2012). They attribute this behavior to an increased induction of polarity in actin stress fibers and traction forces in cells seeded in these narrow channels. However, this technique does not reproduce the subcellular fibrillar architecture of the ECM that can be sensed by migrating cells. To address this limitation, the Reinhart-King's group introduced a micromolding technology to form collagen microtracks that produce a 3D micro-environment which reproduce the physiological structure of native tracks found in proteolytically active cancer cells (Kraning-Rush et al., 2013). Microtracks of collagen have been used to show that adhesion and contractility mechanisms uniquely regulate migration through 3D collagen matrices. Interestingly, migration in collagen microtracks was found to be insensitive to matrix density and also independent of cell-matrix mechano-coupling, which are both important regulators of migration within the 3D matrix (Carey et al., 2015). A migration assay consisting of micro-channels with narrow constrictions has been used to show that nuclear deformation during cell migration leads to transient opening of the nuclear envelope, and that the ESCRT III complex is required to seal rapidly the nucleo-cytoplasmic barrier (Raab et al., 2016). This transient opening of the nuclear membrane causes a mixing of nucleo-cytoplasmic components, that may lead to DNA damages. More recently, large efforts have been made to replicate out-of-plane curvatures, such as tubular structures involved in ductal elongation *in vivo*. Lumens of epithelial cell sheets grown inside narrow microtubes exhibited migration modes that depend on both confinement and curvature levels (Xi et al., 2017).

## Mechanical Probing of Mechanotransduction Signaling Pathways

The characterization of the cells mechanical properties requires to apply well-controlled external forces to induce measurable deformations. The quantification of the mechanical deformation as a function of time and its dependence on the loading frequency allows to determine the viscoelastic behaviors of living cells and tissues. Over the last few years, original methods have been developed to apply well-controlled forces to living cells and tissues. For instance, a parallel plate method based on the bending of glass microplates of calibrated stiffness enables quantifying either passive (i.e., cell deformability) or active (i.e., cell traction) forces. Interestingly, this technique can be combined with confocal microscopy or total internal reflection fluorescence (TIRF) microscopy for studying the dynamics of cell-substrate interactions (Mitrossilis et al., 2010; Fouchard et al., 2014; Bufi et al., 2015).

**Figure 5:**
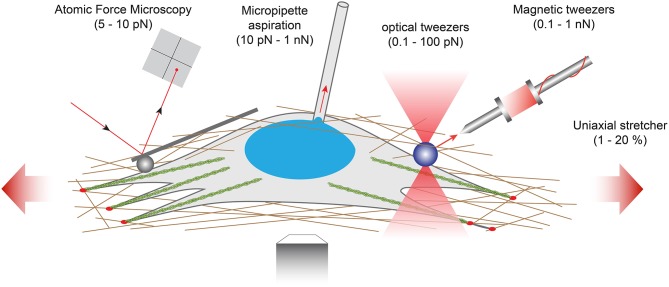
Examples of force-application techniques used to probe the rheological properties of cells or to apply well-defined external loads for studying mechanotransduction pathways. From left to right: atomic force microscopy (AFM), micropipette aspiration, optical tweezers, magnetic tweezers and uniaxial stretcher.

However, the implementation of these techniques in the broader biological community remains challenging due to the inherently multidisciplinary expertise required to conduct and interpret mechanical measurements. Interestingly, some of these force assays can be also used for studying how external forces are transduced into biochemical and functional responses and to identify the corresponding mechanotranduction signaling pathways, in normal or pathological situations (Chen, 2008). In this section, we will review five techniques working at different levels of force and used for interrogating mechanotransduction pathways (Figure 5).

At the lowest force levels, Atomic Force Microscopy (AFM) has progressively emerged as a key platform for mapping the mechanical properties of living biological samples on spatially defined areas ranging from few nanometers to several tens of micrometers. By using AFM to probe fibroblasts seeded on polyacrylamide hydrogels with varying stiffness, Solon *et al*. demonstrated that fibroblasts are able to adapt their own stiffness to match the elastic modulus of its substrate (Solon et al., 2007). The use of cantilevers with a spherical tip protects the cells against damage during force application and enables to use the AFM as a local indenter (Charras and Horton, 2002). This technique was used by Elosegui-Artola et al. to indent the nucleus of fibroblasts and elegantly demonstrate that forces applied to the nucleus translocate the transcription factor YAP to the nucleus (Elosegui-Artola et al., 2017). Recently, AFM has started to be combined with complementary techniques including confocal microscopy, super-resolution microscopy or microfluidic devices to relate the 3D distribution of mechanical responses of biological specimen to their morphologies (Krieg et al., 2019). Indeed, the combination of AFM with additional techniques has been required to investigate the interactions among molecules (Zhou et al., 2017; Bhat et al., 2018). Such combinations of complementary techniques allow to generate multicomplexed mechanical and biochemical data from live cells and tissues in real time.

For larger levels of forces, other techniques have emerged such as optical tweezers (Killian et al., 2018), magnetic tweezers (Kollmannsberger and Fabry, 2007; Sarkar and Rybenkov, 2016), micropipette aspiration (Hochmuth, 2000) or uniaxial stretcher (Figure 5). Optical tweezers (also called optical traps or laser traps) consist of highly focused laser beam that provides an attractive or repulsive force. The laser beam is focused through a microscope objective. The narrowest point of the focused beam, known as the beam waist, contains a very strong electric field gradient. As a consequence, dielectric particles are attracted along the gradient to the region of strongest electric field, which is the center of the beam. The magnitude of the force (typically on the order of 0.1–100 piconewtons) depends on the relative refractive index between the particle and the surrounding medium and allows to physically hold and move micrometer-scale objects, similar to tweezers. Because the trapping force decreases with decreasing object volume, the typical object size ranges from 0.5 to 10 μm in diameter to ensure that objects are trapped efficiently. In addition, the force acting on a bead is dependent on the distance between the laser focal point and the center of the particle. Optical tweezers have been used to study membrane cell elasticity in many cell types such as neurons and red blood cells (Sleep et al., 1999). Optical tweezers were integrated with a microfluidic device to locally apply mechanical tensile and compressive force on single cells, providing an efficient platform for further studies of mechanotransduction in single cells (Honarmandi et al., 2011). More recently, an oscillatory optical trap has been used to apply forces to the cell membrane in the piconewton range. Even if the time-scale of these experiments was very short, this mechanical stimulation produced a local membrane indentation that induces cellular calcium transients, which were observed to be dependent on the stimulus strength and the force pulse frequency (Falleroni et al., 2018). Optical tweezers were usually used to manipulate molecules, but some groups have developed new methodologies based on optical tweezers to probe viscoelastic properties of cells (Yareni et al., 2016). Interestingly, the photo-induced effects caused by laser trapping were found to be negligible, giving the possibility to use optical tweezers for dynamic monitoring of viscoelastic behaviors in living cells and tissues (Lyubin et al., 2012).

Magnetic tweezers rely on the manipulation of paramagnetic beads by applying a controlled magnetic field that exerts pulling forces on the beads. Paramagnetic beads can be chemically functionalized to present adhesion proteins at their surface that can be recognized by the cell cytoskeleton. For instance, Grevesse et al. used fibronectin-coated microbeads to link the cytoskeleton of cortical neurons and probe the mechanical properties of the two main subcellular compartments (soma vs. axon) (Grevesse et al., 2015). Creep experiments revealed two opposite rheological behaviors within cortical neurons: the cell body was soft and characterized by a solid-like response, whereas the neurite compartment was stiffer and viscous-like. The authors suggested that the opposite rheological properties of neuronal microcompartments predict axonal vulnerability in brain injury. The neurite is a mechanosensitive compartment that becomes softer and adopts a pronounced viscous state on soft matrices. Furthermore, they found that local deformations of the cell body induce a significant condensation of chromatin, which results from nuclear shape remodeling that leads to a force-dependent stiffening of the nucleus, providing a robust explanation of the stress stiffening behavior of the soma. Magnetic tweezers were also used to apply high pulsatile forces to fibronectin-coated magnetic beads bound to normal and alpha-actinin depleted cells to demonstrate the role of alpha-actinin in focal adhesion maturation (Roca-Cusachs et al., 2013). Recently, Tajik et al. applied precisely controlled oscillating forces to magnetic beads attached to individual cells. The authors demonstrate that external forces are transmitted to the nucleus, leading to chromatin stretching and changes in gene transcription (Tajik et al., 2016). Indeed, external mechanical stresses were transmitted to the nucleus, where they induced deformation of a bacterial artificial chromosome (BAC) reporter inserted into the cells. By using magnetic tweezers, Tajik and coworkers showed therefore that external forces can modulate the structure of chromatin and the transcription of specific genes (Tajik et al., 2016).

Micropipette aspiration partially aspirates a single cell by applying a subatmospheric pressure through a glass micropipette. This technique was first used on circulating white blood cells (Tsai et al., 1993) and then performed on adherent cells, leading to the estimation of the cell cortical thickness which has been estimated on the order of 0.1 μm (Zhelev et al., 1994).

Other techniques, such as membrane stretching, allow to apply a fixed strain to single cells or a cell sheet by deforming an elastic substrate. The mechanical deformation is thus transmitted to the cells through integrin-related adhesions and involved outside-in mechanotransduction signaling pathways. Silicone elastomers such a poly(dimethylsiloxane), PDMS, are usually used to form elastic membranes. Strain rates commonly vary from 0.1 to 10 Hz with a typical strain percentage ranging from 1 to 30% to keep the membrane deformation in the linear elastic regime. Uniaxial or biaxial strain field can be applied, depending on the complexity of the strain device. One dimensional (1D), or uniaxial strain, corresponds to the membrane stretch in one direction. In this configuration, the two free edges must be constrained to avoid compression in the direction perpendicular to the strain as a result of the Poisson effect. In two-dimensional (2D) strain devices, a uniform biaxial deformation is obtained from the stretching of a thin elastic membrane. Membrane stretching presents the advantage of being applicable on 2D tissues, providing an average readout from millions of cells. In addition, PDMS membranes are optically transparent and could be prepared with thicknesses ranging from 50 to 250 μm, which are particularly well-suited to perform live stretching experiments on an inverted microscope. Uniaxial stretching experiments have been performed to mimic the deformation of brain tissues during traumatic events. Indeed, it was found that traumatic stimulations of integrins are an important etiological contributor to mild Traumatic Brain Injury (mTBI). Cultures of cortical neurons were stretched with an abrupt one-dimensional strain to reproduce *in vitro* TBI. Stretching experiments revealed that the Rho signaling pathway can be activated through integrins and may contribute to the diffuse axonal injury reported in mTBI (Hemphill et al., 2011). By integrating a cell-stretching assay with micropillars, Shao and coworkers found a cell shape-dependent mechanotransduction process in stretched vascular endothelial cells. Combining experiments with theoretical modeling, the authors showed that the global architecture of contractile actomyosin filaments is a key determinant of the mechanotransduction process under uniaxial stretch (Shao et al., 2014).

## Measuring Quantitatively Protrusive and Contractile Forces

Actin filaments (AFs) are semiflexible polymers with a persistence length of ~17 μm (Gittes et al., 1993), which is defined as the distance over which the filament is bent by thermal forces (Morse, 1998). AFs are ~7 nm in diameter, functionally polar in nature and built from dimer pairs of globular actin monomers. Actin polymerization produces most of the driving force required for membrane protrusion (Parekh et al., 2005). When the end of an AF is exposed to a concentration of monomeric actin that is above its critical concentration (Cc), the filament end binds monomers and grows by polymerization. Conversely, when the concentration of monomeric actin is below Cc, monomers detach from the filament end, and the actin filament shrinks by depolymerization. These two different critical actin concentrations are, respectively localized at both opposing ends of the filament, leading to the asymmetrical growth of AFs. When the actin monomer concentration is between the two critical concentrations, only the plus-end grows while the minus-end shrinks. This process allows to maintain a roughly constant length of the filament and is known as “treadmilling” (Wilson et al., 2010).

**Figure 6:**
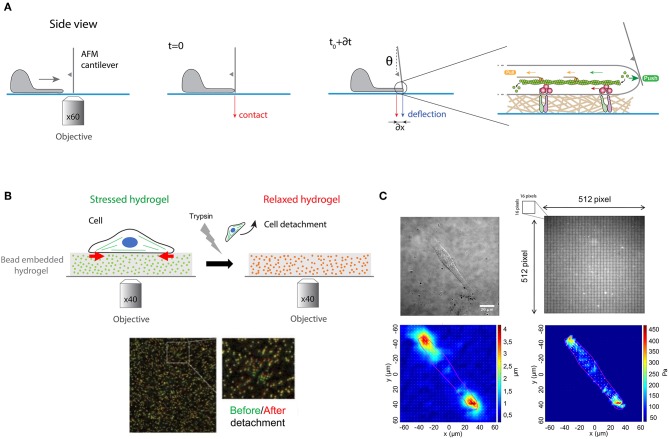
**(A)** Protrusive forces exerted by the lamellipodia of migrating cells can be quantified by measuring the deflection of an AFM cantilever over time. A cantilever of calibrated spring constant is placed vertically perpendicular to the surface in front of the leading edge and its vertical position is measured optically over time. The polymerization of actomyosin filaments exerts a load force that pushes on the cell membrane that deflects the cantilever. **(B)** Traction force microscopy (TFM) measures the contractile forces exerted by adherent cells on an elastic hydrogel that contains fluorescent embedded beads of ~200 nm in diameter. Cell spreads on the hydrogel, leading to its contraction (stressed state in green). After trypsin detachment, the contractile stress is released, and the fluorescent beads relax (in red). The differences of bead position between stressed and relaxed states serve as markers to visualize the hydrogel deformation in a 2D plane. **(C)** Gel deformations are estimated using a Fourier-based difference-with-interpolation image analysis. To characterize the contractile forces of each cell, the elastic strain energy stored in the polyacrylamide gel due to cell tractions is calculated as the product of local tractions and deformations, integrated over the spreading area of the cells. The scale bar is 20 μm.

To test this experimentally, Prass and coworkers introduced a very elegant way to directly measure the protrusive forces exerted by the leading edge (Prass et al., 2006). Briefly, an AFM cantilever of calibrated spring constant was placed vertically perpendicular to the substrate surface in front of the leading edge (Figure 6A). The load force applied by a migrating cell on the advancing leading edge exerts in response an equal and opposite protrusive force to the cantilever. The vertical position of the cantilever was measured optically over time. Knowing the spring constant of the cantilever and measuring its deflection with time permits estimation of the temporal evolution of the load force. When the lamellipodium touched the cantilever, one can observe an initial increase of forces. The deflection increases linearly with time until the lamellipodium is stalled at higher forces. At this stage, the velocity of the lamellipodia decreases close to zero and the leading-edge stalls. The lamellipodial protrusive force was determined from the stall force, corresponding to the moment where the cantilever stops. The first derivative of the deflection-time curves allows to obtain the cantilever speed vs. time. The deflection of the cantilever provides therefore a direct measure of the protrusive forces exerted by the lamellipodium in the nanonewton range.

The actomyosin cytoskeleton of adherent cells generates contractile forces which are transmitted to the ECM through integrin-based adhesions (Figure 1). Contractile forces are crucial for physiological processes such as embryo morphogenesis or wound healing (DuFort et al., 2011) but also for pathological processes, such as cancer metastasis (Wirtz et al., 2011). Measuring cellular traction forces is therefore critical for a better understanding of the mechanisms involved in inside-out and outside-in mechanotransduction signals. To tackle this problem, Harris and coworkers developed the traction force microscopy (TFM) method (Harris et al., 1980). They showed that the wrinkling of an elastic rubber used as culture surface can be calibrated to assess the magnitude of contractile forces exerted by fibroblasts. However, the nonlinear deformation of silicone elastomers and the low spatial resolution lead to the further development of this technique to improve the resolution and reproducibility of traction force measurements (Dembo and Wang, 1999).

In this context, PAAm hydrogels with embedded fluorescent beads of ~200 nm in diameter has emerged as a robust method to determine quantitatively traction forces exerted by adherent cells (Figures 6B,C). Adherent cells deform the substrate with cellular tractions that have a very small magnitude (pN—nN) and occur across small length scales (nm—μm). Due to their superior optical and mechanical properties, PAAm hydrogels are now considered as the substrate of choice to perform continuous traction force measurements. Indeed, PAAm hydrogels are optically transparent and their mechanical properties are also ideal since they are linearly elastic over a wide range of deformations. Traction forces can be estimated by comparing two images of fluorescent images of fluorescent markers embedded in the elastic hydrogel (Figure 6B). The first image corresponds to the stressed state where the cell is applying traction forces on the substrate, whereas the second corresponds to the fully relaxed state of the substrate. This reference image is obtained by detaching cells with a trypsin treatment. Although TFM was originally conceived to compute a 2D force field exerted by a single cell on a flat substrate (Han et al., 2015), this method has been then successfully extended to multicellular clusters (Trepat et al., 2009). Recently a large effort has been made to compute 3D force fields of cells moving in complex fibrillar 3D microenvironment. Indeed, physiologically the ECM is mostly composed of fibers that behave as non-linear elastic materials. To address this problem 3D traction force fields were computed using synthetic polyethylene glycol (PEG) with matrix metalloprotease (MMP)-cleavable sites (Legant et al., 2010). Interestingly, 3D traction force approaches have shown that the traction force exerted by MDA-MB-231 breast carcinoma cells is independent of ECM concentration and stiffness (Steinwachs et al., 2016). It is important to note that determining the traction field from the displacement field represents a mathematical ill-posed problem that can be solved only by a small number of experts in force-field calculations. This limitation has been addressed by providing efficient open source codes (Schwarz and Soine, 2015). Recently, hydrogels labeled with a high density of fluorescent microspheres of two different colors have been developed (Sabass et al., 2008) to analyze both the distribution and dynamics of traction forces within individual focal adhesions (Plotnikov et al., 2012). In addition, light-based methods have been developed by employing molecular tension sensors, such as Förster resonance energy transfer (FRET) and deoxyribonucleic acid (DNA) (Jurchenko and Salaita, 2015). The combination of TFM with FRET-based molecular force sensors is an efficient tool to decipher the mechanisms of integrin-mediated mechanosensing (Grashoff et al., 2010; Blakely et al., 2014; Zhang et al., 2014; Jurchenko and Salaita, 2015). Moreover, novel strategies using membrane DNA tension probes allow to visualize tensile forces at cell junctions (Zhao et al., 2017).

*In vivo* cells exist within three-dimensional (3D) matrices, however measuring tractions of cells in 3D remains difficult and the nonlinear nature of collagen type I hydrogels, which is already used for 3D cell culture, prevents calculation of traction forces. To address this challenge, vertical cantilevers were molded from silicone elastomers in order to measure forces from tissue constructs composed of few 100 of cells (Legant et al., 2009). This device is now considered as a robust assay to determine contractile forces in cardiac tissues, providing quantitative information about the mechanotransduction signaling pathways that drive tissue formation (Boudou et al., 2012).

## Determining Mechanical and Functional Properties of the Cell Nucleus

The mechanical stability of the nucleus defines its capability of maintaining a three-dimensional shape by minimizing deformations and recovering strain after a mechanical deformation. Interestingly, it has been demonstrated that large nuclear deformations increase the risk of modifying nuclear architecture and may lead to DNA damages and ultimately cell death (Versaevel et al., 2013; Denais et al., 2016).

The quantifications of deformations and recovery of nuclei are required for understanding the mechanisms involved in the nuclear response to forces and in the maintenance of its mechanical stability. To address this challenge, micropipette aspiration has been shown to be an interesting method by providing a robust way to characterize nuclear viscoelastic properties. This technique was used by Guilak and coworkers to demonstrate the individual contribution of each nuclear components. The authors conclude that nuclei behave as a viscoelastic material which is 3–4 times stiffer than the cytoplasm and nearly twice as viscous as the cytoplasm (Guilak et al., 2000). Dahl and coworkers used the micropipette aspiration technique to demonstrate that the nucleus is a stiff organelle that maintains its mechanical integrity at short times, but deforms at longer times (Dahl et al., 2005). Recently, (de-)adhesion kinetics on micropatterned substrates have been introduced as a robust assay for studying cellular and nuclear mechanics. Interestingly, this non-invasive technique can be extended to high-throughput assays for screening pharmacological candidates (Versaevel et al., 2017).

**Figure 7:**
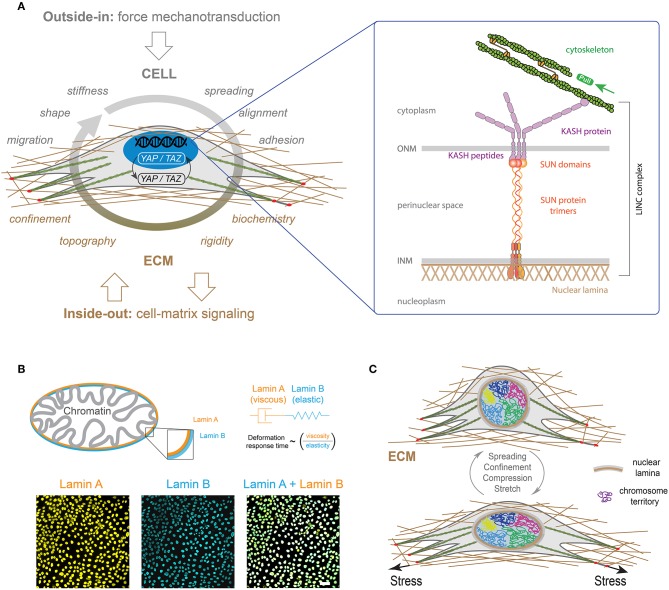
**(A)** LINC complexes are made of the inner nuclear membrane (INM), SUN protein trimers, the outer nuclear membrane (ONM) and KASH proteins. KASH protein mediates interactions with actin cables through Nesprin 2. **(B)** A-type and B-type lamins (orange and blue, respectively) form individual polymer networks which are juxtaposed on the inside of the nuclear envelope. Both lamin types are found at the interface between chromatin and the cytoskeleton (Swift and Discher, 2014). The mechanical properties of the lamina can be described by viscoelastic models based on the combination of elastic (spring-like) springs and viscous (flowing) dahspots. The characteristic deformation time of the lamina is therefore related to the ratio of the viscous part (i.e., lamin A) on the elastic one (i.e., lamin B) (Swift et al., 2013). Typical images of nuclei of Madin-Darby Canine Kidney (MDCK) cells stained for lamin A (in orange) and lamin B (in blue). The scale bar is 10 μm. **(C)** Differential mechanical forces are applied on the nucleus, such as actin contractile forces or microtubules compressive forces, through LINC complexes. The three-dimensional organization of chromosomes is modulated by these mechanical signals, leading to new gene expression programs (Uhler and Shivashankar, 2017).

It has been shown that genome expression is affected by spatial positioning and chromatin motions (Misteli, 2004) but also by nuclear compartmentalization, and other factors that are all physically contained by the nuclear envelope (NE). The NE separates the chromatin from the cytoplasm and contains the peripheral protein lamina (Gruenbaum et al., 2003), which is attached by nuclear membrane proteins that stabilize the envelope and provide sites for chromatin binding and organization (Figure 7A) (Burke and Stewart, 2002).

The overall nuclear stiffness is mainly established by the remodeling of lamina and chromatin. It is therefore important to decipher their individual roles for a better understanding of the nuclear mechanics and the regulation of cellular functions. It has been shown that chromatin controls the resistance to small deformations, while lamina dictates the nuclear strain stiffening that dominates resistance to large deformations (Stephens et al., 2017). In a first approximation, the mechanical response of the lamina can be described by a Maxwell model that combines in series a purely elastic spring with a purely viscous damper. Recent works have shown that B-type lamins contribute primarily to the elastic response, whereas A-type lamins contribute to the viscosity (Figure 7B) (Swift et al., 2013). As a consequence, increasing A-type lamins relative to B-type lamins leads to slower nuclear deformation under stress (Swift et al., 2013). Recently, the loss of A-type lamins in human dermal fibroblasts was observed to correspond to large stress fibers and high traction forces, suggesting a role of A-type lamins in the balance between cytoskeletal tension and cell-substrate adhesions, which may contribute to mechanosensing defects as observed in laminopathies (Corne et al., 2017). It is now clear that there is a global connection between the physico-chemical properties of micro-environment and gene expression. Indeed, integrins at the focal adhesions link the cytoskeleton to the ECM proteins, which is in close interaction with the nuclear membrane through LINC complex (Figure 7A). Using Structured Illumination Microscopy (SIM), it has been shown that nuclear indentations are generated by accumulated tension in apical actin stress fibers that deform the nuclear lamina. Indeed a local enrichment of LINC complexes has been observed at indented nuclear zones where apical actin fibers are anchored to the nuclear lamina. In addition, the deep deformations of the nuclear envelope indices the formation of segregated domains of condensed chromatin (Versaevel et al., 2014a). Finally, Lamina-associated domains (LADs) along DNA enables the interactions between chromosomes and nuclear membrane. This global structure suggests that modifications of the ECM can modulate the regulation of the genome architecture and cell-fate decisions (Shivashankar, 2019).

Cell shape changes have been found to modulate nuclear shape, DNA expression (Versaevel et al., 2012), histone acetylation and gene expression profile (Jain et al., 2013). Inside nuclei, DNA is packed into less condensed euchromatin and more condensed heterochromatin, that both form chromosomes. The spatial organization of chromosomes into distinct chromosome territories inside the nucleus is crucial for regulation of gene expression (Figure 7C) (Bickmore and van Steensel, 2013). Interestingly, the reorganization of these territories has been observed during differentiation (Boney and Cavalli, 2016). In view of the linkage between the cellular micro-environment and the nucleus through the cytoskeleton and specific connectors, the question of the effect of external mechanical stresses applied to the cell on the spatial organization of chromatin was raised. To address this question, Wang et al. used fluorescence hybridization to demonstrate that the repositioning of chromosomes correlates with gene expression that were found to be cell geometry dependent (Wang et al., 2017). More recently, Roy et al. demonstrated the reversibility of the chromosome territories transitions by reprogramming fibroblasts into stem cell-like cells through laterally confined growth (Roy et al., 2018). Growing evidence suggests that a mechanical load on the nucleus drives the nuclear translocation of YAP by modulating molecular transport through nuclear pores (Figure 7A) (Elosegui-Artola et al., 2017). In addition, the stiffening of the ECM allows to connect the cytoskeleton and the nucleus, allowing the propagation of forces from focal adhesions to the nuclear envelope.

## Optogenetic Methods to Interrogate Mechanotransduction Pathways

**Figure 8:**
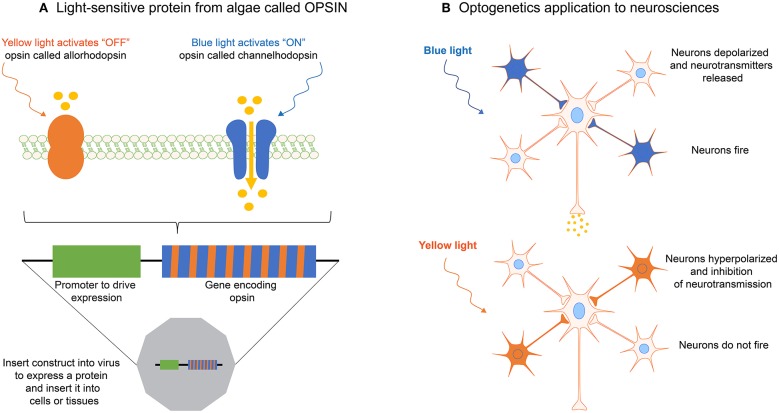
**(A)** The optogenetic principle is based on light-sensitive proteins from algae called opsins. **(B)** Opsins can be expressed in single cells, cell clusters or tissues by using transfection with a gene construct inserted in viruses. By using different wavelengths, optogenetics allows to activate or inhibit for instance neurotransmission, or specific mechanotransduction signaling pathways in living cells, such as neurons.

Optogenetics is a rapidly evolving technology that aims to control optically and precisely specific events in genetically targeted living cells and tissues. Optogenetic methods allow to deliver an optical control at high temporal (millisecond-scale) and spatial resolution in physiological or pathological situations (Deisseroth, 2011). The principle of optogenetics uses the heterologous expression of light-sensitive microbial membrane proteins, called opsins, to induce optically a cell depolarization or silencing on a millisecond time scale (Figure 8A). Optogenetics combines therefore optics, genetics and bioengineering to either stimulate or inhibit cellular activity via light-sensitive opsins (Figure 8B) (Nagel et al., 2002, 2003).

In 1979 Francis Crick described one of the major challenges facing neuroscience by the need to control one cell type in the brain while leaving other cell types unaltered. Since electrodes cannot be used to precisely target defined cells, Crick envisioned that light might be used to control and monitor the activity of genetically defined neuronal populations. However, it took more than 30 years for neuroscientists to develop the first approaches for optogenetics. In the early 2000s, Zemelman and Miesenbock (Sloan- Kettering Cancer center, New York) (Zemelman et al., 2003) and Trauner, Kramer and Isacoff (University of California, Berkeley) (Banghart et al., 2004) considered alternative strategies based on cascades or combination of different genes. In 2005, Deisseroth and coworkers used a light-sensitive microbial protein, Channelrhodopsin-2 (ChR2), expressed in neurons to activate neurons with light pulses in a temporally precise manner (Boyden et al., 2005). The current optogenetics tool box opens the door to experiments where neuronal activity can be controlled in real time.

Although it arose from neurosciences, optogenetics has started to address recently open questions about mechanotransduction mechanisms in various cell types. For instance, Bruegman *et al*. showed that optogenetic stimulations of skeletal muscles expressing the light-sensitive channel ChR2 can generate large forces, which could be useful for studying mechanotransduction signaling pathways in muscle cells (Bruegmann et al., 2015). More recently, Valon and coworkers reported the up- and down- regulation of contractile forces with optogenetic tools working at high spatiotemporal accuracy (Valon et al., 2017). The authors found a rapid increase of cellular traction forces in response to the translocation of RhoA activator ARHGEF11 to CRY2-mCherry (optoGEF-RhoA). Changes in cell contractility were found to be related to modifications in the transcriptional regulator YAP, demonstrating the ability of optogenetic approach to control mechanotransduction signaling pathways. More recently, Baaske and coworkers reported an optogenetic system based on an integrin engineered with a phytochrome-interacting factor domain (OptoIntegrin) and a red light-switchable phytochrome B-functionalized matrix (OptoMatrix) (Baaske et al., 2019). This receptor-ligand pair enables a reversible optogenetic control of integrin-matrix interaction, as well as the controlled activation of downstream mechanosensory signaling pathways.

## Summary and Future Perspectives

Novel techniques developed to probe cellular forces have reported a wide range of mechanisms acting over multiple length scales (from molecular forces to supra-cellular force patterns). As a consequence, physical forces cannot be only considered as basic switches of mechanotransduction signals, but as the key mechanism to propagate signals between cells. Interestingly, recent technological developments allow to study the molecular mechanisms used by cellular forces to alter gene activities by modulating the conversion of mechanical stimuli into biochemical signals. Advancements in force measurement methods will therefore permit to address many remaining open questions surrounding cell-substrate but also cell-cell interactions, such as cadherins. For instance, understanding how mechanical tension exerted on cadherins is converted into biochemical signals and how this signaling in turn leads to changes in cell expression remains an open question in cell biology.

The understanding of the molecular mechanisms involved in outside-in and inside-out mechanotransduction signaling pathways requires to elaborate integrated strategies combining super resolution fluorescence microscopy (e.g., stimulated emission depletion, STED—photo-activated localization microscopy, PALM—stochastic optical reconstruction microscopy, STORM) with biophysical probes and multipatterning of proteins. In addition to these combined imaging techniques, FRET biosensors could be applied to examine force transmission across the cytoskeleton to nuclear envelope proteins, chromatin remodeling or mechanically induced changes within the nucleus. FRET between fluorophores of a single type, known as homoFRET, is a promising method to visualize and quantitatively measure changes in protein ratios upon force application, based on the signal produced when molecules labeled with enhanced green fluorescent protein, such as G-actin, assemble into actin filaments.

A further challenge should be devoted to a better understanding of how ECM sensing can activate specific transcription factors and translocates them to the cell nucleus. Indeed, it remains unclear whether chromosome configurations can be altered in response to the modifications of the mechanical properties of the nucleus that can be modulated by changes in the ECM physico-chemical properties. Furthermore, several aspects of Piezo channels must be addressed in the near future to determine the role of specific mechanotransduction processes in regulating physiological and pathological processes. For instance, it will be important to decouple the role of Piezo 1 and 2 that makes them such versatile mechanosensors. In addition, questions about conformational changes leading to channel (in-)activation remain to be answered.

Finally, a major technical challenge in cellular mechanotransduction concerns the development of synthetic models of the stem cell niche to manipulate the biophysical and biochemical properties of the stem cell microenvironment. Indeed, the discovery of induced pluripotent stem (iPS) cells as patient-specific stem cells represents a breakthrough for the basic cell biology and new therapies. Understanding how stem cell behavior can be regulated by mechanical forces can provide fundamental insights for the design of artificial niches for regenerative therapies. In this context, we envision exciting technical developments for the light-controlled activation of cellular forces, dynamic organoid systems and synthetic niches with a spatiotemporally controlled release of proteins and growth factors. Smart stem cell niches that integrate the control of material properties (stiffness, topography, etc.) and protein patterning to recapitulate cell-cell and cell-matrix interactions is required to identify the ECM cues which are relevant to niche-like regulation of stem cell fate. By guiding collections of stem cells that can assemble in 3D, organ-on-a-chip platforms represent a valuable technology to form realistic *in vitro* models of organ-level systems required to interrogate mechanotransduction pathways in stem cells but also to realize a precision medicine approach by testing important differences in varied patient cohorts.

## Author Contributions

DM and SG designed the content of the article. All authors performed literature survey, prepared the figures and wrote the article. All authors edited and reviewed the article before submission.

### Conflict of Interest Statement

The authors declare that the research was conducted in the absence of any commercial or financial relationships that could be construed as a potential conflict of interest.

## References

[B1] Annabi N. Tamayol A. Uquillas J. A. Akbari M. Bertassoni L. E. Cha C.. (2014). 25th anniversary article: rational design and applications of hydrogels in regenerative medicine. Adv. Mater. Weinheim. 26, 85–123. 10.1002/adma.201303233 24741694 PMC3925010

[B2] Baaske J. Mülhäuser W. W. D. Sasha Yousefi O. Zanner S. Radziwill G. Hörner M.. (2019). Optogenetic control of integrin-matrix interaction. Commun. Biol. 2:15. 10.1038/s42003-018-0264-7 30652127 PMC6325061

[B3] Baker B. M. Trappmann B. Wang W. Y. Sakar M. S. Kim I. L. Shenoy V. B.. (2015). Cell-mediated fibre recruitment drives extracellular matrix mechanosensing in engineered fibrillar microenvironments. Nat. Mater. 14, 1262–1268. 10.1038/nmat4444 26461445 PMC4654682

[B4] Banghart M. Borges K. Isacoff E. Trauner D. Kramer R. H. (2004). Light-activated ion channels for remote control of neuronal firing. Nat. Neurosci. 7, 1381–1386. 10.1038/nn1356 15558062 PMC1447674

[B5] Bauer A. Gu L. Kwee B. Li W. A. Dellacherie M. Celiz A. D.. (2017). Hydrogel substrate stress-relaxation regulates the spreading and proliferation of mouse myoblasts. Acta Biomater. 62, 82–90. 10.1016/j.actbio.2017.08.041 28864249 PMC5641979

[B6] Bhat S. V. Sultana T. Körnig A. McGrath S. Shahina Z. Dahms T. E. S. (2018). Correlative atomic force microscopy quantitative imaging-laser scanning confocal microscopy quantifies the impact of stressors on live cells in real-time. Sci. Rep. 8:8305. 10.1038/s41598-018-26433-1 29844489 PMC5973941

[B7] Bickmore W. A. van Steensel B. (2013). Genome architecture: domain organization of interphase chromosomes. Cell 152, 1270–1284. 10.1016/j.cell.2013.02.001 23498936

[B8] Blakely B. L. Dumelin C. E. Trappman B. McGregor L. M. Choi C. K. Anthony P. C.. (2014). A DNA-based molecular probe for optically reporting cellular traction forces. Nat. Methods 11, 1229–1232. 10.1038/nmeth.3145 25306545 PMC4247985

[B9] Boney B. Cavalli G. (2016). Organization and function of the 3D genome. Nat. Rev. Genet. 17, 661–678. 10.1038/nrg.2016.112 27739532

[B10] Boudou T. Legant W. R. Mu A. Borochin M. A. Thavandiran N. Radisic M.. (2012). A microfabricated platform to measure and manipulate the mechanics of engineered cardiac microtissues. Tissue Eng. Part A 18, 910–919. 10.1089/ten.tea.2011.0341 22092279 PMC3338105

[B11] Boyden E. S. Zhang F. Bamberg E. Nagel G. Deisseroth K. (2005). Millisecond-timescale, genetically targeted optical control of neural activity. Nat. Neurosci. 8, 1263–1268. 10.1038/nn1525 16116447

[B12] Bruegmann T. van Bremen T. Vogt C. C. Send T. Fleischmann B. K. Sasse P. (2015). Optogenetic control of contractile function in skeletal muscle. Nat. Commun. 2:7153 10.1038/ncomms8153 PMC4475236 26035411

[B13] Bryant S. J. Cuy J. L. Hauch K. D. Ratner B. D. (2007). Photo-patterning of porous hydrogels for tissue engineering. Biomaterials 28, 2978–2986. 10.1016/j.biomaterials.2006.11.033 17397918 PMC1950475

[B14] Bufi N. Durand-Smet P. Asnacios A. (2015). Single-cell mechanics: the parallel plates technique. Methods Cell Biol. 125, 187–209. 10.1016/bs.mcb.2014.11.002 25640430

[B15] Burke B. Stewart C. L. (2002). Life at the edge: the nuclear envelope and human disease. Nat. Rev. Mol. Cell Biol. 3, 575–585. 10.1038/nrm879 12154369

[B16] Carey S. P. Rahman A. Kraning-Rush C. M. Romero B. Somasegar S. Torre O. M.. (2015). Comparative mechanisms of cancer cell migration through 3D matrix and physiological microtracks. Am. J. Physiol. Cell Physiol. 308, C436–C447. 10.1152/ajpcell.00225.2014 25500742 PMC4360026

[B17] Carter S. B. (1967). Haptotactic islands – a method of confining single cells to study individual cell reactions and clone formation. Exp. Cell Res. 48, 189–193. 10.1016/0014-4827(67)90298-4 4862713

[B18] Chabria M. Hertig S. Smith M. L. Vogel V. (2010). Stretching fibronectin fibres disrupts binding of bacterial adhesins by physically destroying an epitope. Nat. Commun. 1:135. 10.1038/ncomms1135 21139580 PMC3105298

[B19] Charras G. T. Horton M. A. (2002). Single cell mechanotransduction and its modulation analyzed by atomic force microscope indentation. Biophys. J. 82, 2970–2981. 10.1016/S0006-3495(02)75638-5 12023220 PMC1302085

[B20] Charrier E. E. Pogoda K. Wells R. G. Janmey P. (2018). Control of cell morphology and differentiation by substrates with independently tunable elasticity and viscous dissipation. Nat. Comm. 9:449. 10.1038/s41467-018-02906-9 29386514 PMC5792430

[B21] Chauduri O. Gu L. Darnell M. Klumpers D. Bencherif S. A. Weaver J. C. (2015). Substrate stress relaxation regulates cell spreading. Nat. Commun. 6:6365 10.1038/ncomms7365 PMC4518451 25695512

[B22] Chauduri O. Gu L. Klumpers D. Darnell M. Bencherif S. A. Weaver J. C. (2016). Hydrogels with tunable stress relaxation regulate stem cell fate and activity. Nat. Mater. 15, 326–334. 10.1038/nmat4489 26618884 PMC4767627

[B23] Chen C. S. (2008). Mechanotransduction - a field pulling together? J. Cell Sci. 121, 285–3292. 10.1242/jcs.023507 18843115

[B24] Chen C. S. Mrksich M. Huang S. Whitesides G. M. Ingber D. E. (1997). Geometric control of cell life and death. Science 276, 425–428. 10.1126/science.276.5317.1425 9162012

[B25] Chen T. Callan-Jones A. Fedorov E. Ravasio A. Brugues A. Ong H. T.. (2019). Large-scale curvature sensing by directional actin flow drives cellular migration mode switching. Nat. Phys. 15, 393–402. 10.1038/s41567-018-0383-6 30984281 PMC6456019

[B26] Chu S. H. Lo L. H. Lai R. L. Yang T. T. Weng R. R. Liao J. C.. (2019). A microfluidic device for in situ fixation and super-resolved mechanosensation studies of primary cilia. Biomicrofluidics 13:014105. 10.1063/1.5081756 30867876 PMC6404955

[B27] Coppée S. Gabriele S. Jonas A. Jestin J. Damman P. (2011). Influence of Chain Interdiffusion between Immiscible Polymers on Dewetting Dynamics. Soft Matter 7, 9951–9955. 10.1039/c1sm05486d

[B28] Corne T. D. J. Sieprath T. Vandenbussche J. Mohammed D. te Lindert M. Gabriele S.. (2017). Deregulation of focal adhesion formation and cytoskeletal tension due to loss of A-type lamins. Cell Adh. Migr. 11, 447–463. 10.1080/19336918.2016.1247144 27791462 PMC5810761

[B29] Coste B. Mathur J. Schmidt M. Early T. J. Ranade S. Petrus M. J.. (2010). Piezo1 and Piezo2 are essential components of distinct mechanically activated cation channels. Science 330, 55–60. 10.1126/science.1193270 20813920 PMC3062430

[B30] Dahl K. N. Engler A. J. Pajerowski J. D. Discher D. E. (2005). Power-law rheology of isolated nuclei with deformation mapping of nuclear substructures. Biophys. J. 89:2855. 10.1529/biophysj.105.062554 16055543 PMC1366783

[B31] Damljanovic V. Lagerholm B. C. Jacobson K. (2005). Bulk and micropatterned conjugation of extracellular matrix proteins to characterized polyacrylamide substrates for cell mechanotransduction assays. BioTechniques 39, 847–851. 10.2144/000112026 16382902

[B32] Deisseroth K. (2011). Optogenetics. Nat. Methods 8, 26–29. 10.1038/nmeth.f.324 21191368 PMC6814250

[B33] Dembo M. Wang Y. L. (1999). Stresses at the cell-to-substrate interface during locomotion of fibroblasts. Biophys. J. 76, 2307–2316. 10.1016/S0006-3495(99)77386-8 10096925 PMC1300203

[B34] Denais C. M. Gilbert R. M. Isermann P. McGregor A. L. te Lindert M. Weigelin B.. (2016). Nuclear envelope rupture and repair during cancer cell migration. Science 352, 353–358. 10.1126/science.aad7297 27013428 PMC4833568

[B35] Dong Y. Jin G. Hong Y. Zhu H. Lu T. J. Xu F.. (2018). Engineering the cell microenvironment using novel photoresponsive hydrogels. ACS Appl. Mater. Interfaces 10, 12374–12389. 10.1021/acsami.7b17751 29537822

[B36] Duclos G. Blanch-Mercader C. Yashunsky V. Salbreux G. Joanny J. F. Prost J.. (2018). Spontaneous shear flow in confined cellular nematics. Nat. Phys. 14, 728–732. 10.1038/s41567-018-0099-7 30079095 PMC6071846

[B37] Duclos G. Garcia S. Yevick H. G. Silberzan P. (2014). Perfect nematic order in confined monolayers of spindle-shaped cells. Soft Matter 10, 2346–2353. 10.1039/C3SM52323C 24623001

[B38] DuFort C. C. Paszek M. J. Weaver V. M. (2011). Balancing forces: architectural control of mechanotransduction. Nat. Rev. Mol. Cell Biol. 12, 308–319. 10.1038/nrm3112 21508987 PMC3564968

[B39] Elosegui-Artola A. Andreu I. Beedle A. E. M. Lezamiz A. Uroz M. Kosmalska A. J.. (2017). Force triggers YAP nuclear entry by regulating transport across nuclear pores. Cell 171, 1397–1410. 10.1016/j.cell.2017.10.008 29107331

[B40] Engler A. J. Sen S. Sweeney H. L. Discher D. E. (2006). Matrix elasticity directs stem cell lineage specification. Cell 126, 677–689. 10.1016/j.cell.2006.06.044 16923388

[B41] Falleroni F. Torre V. Cojoc D. (2018). Cell mechanotransduction with piconewton forces applied by optical tweezers. Front. Cell. Neurosci. 12:130 10.3389/fncel.2018.00130 29867363 PMC5960674

[B42] Fink J. Carpi N. Betz T. Betard A. Chebah M. Azioune A.. (2011). External forces control mitotic spindle positioning. Nat. Cell Biol. 13, 771–778. 10.1038/ncb2269 21666685

[B43] Fouchard J. Bimbard C. Bufi N. Durant-Smet P. Proag A. Richert A.. (2014). Three-dimensional cell body shape dictates the onset of traction force generation and growth of focal adhesions. Proc. Natl. Acad. Sci. U.S.A 111, 13075–13080. 10.1073/pnas.1411785111 25157134 PMC4246942

[B44] Gittes F. Mickey B. Nettleton J. Howard J. (1993). Flexural rigidity of microtubules and actin filaments measured from thermal fluctuations in shape. J. Cell Biol. 120, 923–934. 10.1083/jcb.120.4.923 8432732 PMC2200075

[B45] Grashoff C. Hoffman B. D. Brenner M. D. Zhou R. Parsons M. Yang M. T.. (2010). Measuring mechanical tension across vinculin reveals regulation of focal adhesion dynamics. Nature 466, 263–267. 10.1038/nature09198 20613844 PMC2901888

[B46] Grevesse T. Dabiri B. E. Parker K. K. Gabriele S. (2015). Opposite rheological properties of neuronal microcompartments predict axonal vulnerability in brain injury. Sci. Rep. 5:9475. 10.1038/srep09475 25820512 PMC4377573

[B47] Grevesse T. Versaevel M. Circelli G. Desprez S. Gabriele S. (2013). A simple route to functionalize polyacrylamide hydrogels for the independent tuning of mechanotransduction cues. Lab Chip 13:777. 10.1039/c2lc41168g 23334710

[B48] Grevesse T. Versaevel M. Gabriele S. (2014). Preparation of Hydroxy-PAAm Hydrogels for decoupling the effects of mechanotransduction cues. J. Vis. Exp. 90:e51010 10.3791/51010 PMC4828013 25225964

[B49] Gruenbaum Y. Goldman R. D. Meyuhas R. Mills E. Margalit A. Fridkin A.. (2003). The nuclear lamina and its functions in the nucleus. Int. Rev. Cytol. 226, 1–62. 10.1016/S0074-7696(03)01001-5 12921235

[B50] Guilak F. Tedrow J. R. Burgkart R. (2000). Viscoelastic properties of the cell nucleus. Biochem. Biophys. Res. Commun. 269, 781–786. 10.1006/bbrc.2000.2360 10720492

[B51] Han S. J. Oak Y. Groisman A. Danuser G. (2015). Traction microscopy to identify force modulation in subresolution adhesions. Nat. Methods 12, 653–656. 10.1038/nmeth.3430 26030446 PMC4490115

[B52] Harris A. (1973). Behavior of cultured cells on substrata of variable adhesiveness. Exp. Cell Res. 77, 285–297. 10.1016/0014-4827(73)90579-X 4570353

[B53] Harris A. K. Wild P. Stopak D. (1980). Silicone rubber substrata: a new wrinkle in the study of cell locomotion. Science 208, 177–179. 10.1126/science.6987736 6987736

[B54] Hemphill M. A. Dabiri B. E. Gabriele S. Kerscher L. Franck C. Goss J. A.. (2011). A possible role for integrin signaling in Diffuse Axonal Injury. PLoS ONE 6:e22899. 10.1371/journal.pone.0022899 21799943 PMC3142195

[B55] Hochmuth R. M. (2000). Micropipette aspiration of living cells. J. Biomech. 33, 15–22. 10.1016/S0021-9290(99)00175-X 10609514

[B56] Honarmandi P. Lee H. Lang M. J. Kamm R. D. (2011). A microfluidic system with optical laser tweezers to study mechanotransduction and focal adhesion recruitment. Lab Chip. 11, 684–694. 10.1039/C0LC00487A 21152510

[B57] Humphrey J. D. Dufresne E. R. Schwartz M. A. (2014). Mechanotransduction and extracellular matrix homeostasis. Nat. Rev. Mol. Cell Biol. 12, 802–812. 10.1038/nrm3896 PMC4513363 25355505

[B58] Humphries M. J. (1990). The molecular basis and specificity of integrin-ligand interactions. J. Cell Sci. 97, 585–592. 2077034 10.1242/jcs.97.4.585

[B59] Jaalouk D. E. Lammerding J. (2009). Mechanotransduction gone awry. Nat. Rev. Mol. Cell Biol. 10, 63–73. 10.1038/nrm2597 19197333 PMC2668954

[B60] Jain N. Venkatesan Iyer K. Kumar A. Shivashankar G. V. (2013). Cell geometric constraints induce modular gene-expression patterns via redistribution of HDAC3 regulated by actomyosin contractility. Proc. Natl. Acad. Sci. U.S.A. 110, 11349–11354. 10.1073/pnas.1300801110 23798429 PMC3710882

[B61] Jurchenko C. Salaita K. S. (2015). Lighting up the force: investigating mechanisms of mechanotransduction using fluorescent tension probes. Mol. Cell. Biol. 35, 2570–2582. 10.1128/MCB.00195-15 26031334 PMC4524122

[B62] Karp G. (2015). Cell and Molecular Biology: Concepts and Experiments. New York, NY: John Wiley & Sons, Inc.

[B63] Katta S. Krieg M. Goodman M. B. (2015). Feeling force: physical and physiological principles enabling sensory mechanotransduction. Annu. Rev. Cell Dev. Biol. 31, 347–371. 10.1146/annurev-cellbio-100913-013426 26566115

[B64] Killian J. L. Ye F. Wang M. D. (2018). Optical Tweezers: a force to be reckoned with Cell 175, 1445–1448. 10.1016/j.cell.2018.11.019 30500527

[B65] Klotzsch E. Smith M. L. Kubow K. E. Muntwyler S. Little W. C. Beyeler F.. (2009). Fibronectin forms the most extensible biological fibers displaying switchable force-exposed cryptic binding sites. Proc. Natl. Acad. Sci. U.S.A. 106, 18267–1872. 10.1073/pnas.0907518106 19826086 PMC2761242

[B66] Kloxin A. M. Kasko A. M. Salinas C. N. Anseth K. S. (2009). Photodegradable hydrogels for dynamic tuning of physical and chemical properties. Science 324, 59–63 10.1126/science.1169494 19342581 PMC2756032

[B67] Kollmannsberger P. Fabry B. (2007). High-Force Magnetic tweezers with force feedback for biological applications. Rev. Sci. Instrum. 78:14301. 10.1063/1.2804771 18052492

[B68] Kraning-Rush C. M. Carey S. P. Lampi M. C. Reinhart-King C. A. (2013). Microfabricated collagen tracks facilitate single cell metastatic invasion in 3D. Integr. Biol. 5, 606–616. 10.1039/c3ib20196a 23388698 PMC3601578

[B69] Krieg M. Fläschner G. Alsteens D. Gaub B. M. Roos W. H. Wuite G. J. L. (2019). Atomic force microscopy-blased mechanobiology. Nat. Rev. Phys. 1, 41–57. 10.1038/s42254-018-0001-7

[B70] Kubow K. E. Vukmirovic R. Zhe L. Klotzsch E. Smith M. L. Gourdon D.. (2015). Mechanical forces regulate the interactions of fibronectin and collagen I in extracellular matrix. Nat. Commun. 6:8026. 10.1038/ncomms9026 26272817 PMC4539566

[B71] Lantoine J. Grevesse T. Villers A. Delhaye G. Mestdagh C. Versaevel M.. (2016). Matrix stiffness modulates formation and activity of neuronal networks of controlled architectures. Biomaterials 89:14e24. 10.1016/j.biomaterials.2016.02.041 26946402

[B72] Legant W. R. Miller J. S. Blakely B. L. Cohen D. M. Genin G. M. Chen C. S. (2010). Measurement of mechanical tractions exerted by cells in three-dimensional matrices. Nat. Methods 7, 969–971. 10.1038/nmeth.1531 21076420 PMC3056435

[B73] Legant W. R. Pathak A. Yang M. T. Deshpande V. S. McMeeking R. M. Chen C. S. (2009). Microfabricated tissue gauges to measure and manipulate forces from 3D microtissues. Proc. Natl. Acad. Sci. U.S.A. 106, 10097–10102. 10.1073/pnas.0900174106 19541627 PMC2700905

[B74] Liu W. Jawerth L. M. Sparks E. A. Falvo M. R. Hantgan R. R. Superfine R.. (2006). Fibrin fibers have extraordinary extensibility and elasticity. Science 313:634. 10.1126/science.1127317 16888133 PMC1950267

[B75] Liu X. M. Yang B. Wang Y. L. (2005). Photoisomerisable cholesterol derivatives as photo-trigger of liposomes: effect of lipid polarity, temperature, incorporation ratio, and cholesterol. Biochim. Biophys. Acta 1720, 28–34. 10.1016/j.bbamem.2005.10.016 16368070

[B76] Lueckgen A. Garske D. S. Ellinghaus A. Desai R. M. Stafford A. G. Mooney D. J.. (2018). Hydrolytically-degradable click-crosslinked alginate hydrogels. Biomaterials 181, 189–198. 10.1016/j.biomaterials.2018.07.031 30086448

[B77] Lyubin E. V. Khokhlova M. D. Skryabina M. N. Fedyanin A. A. (2012). Cellular viscoelasticity probed by active rheology in optical tweezers. J. Biomed. Opt. 17:101510. 10.1117/1.JBO.17.10.101510 23223986

[B78] Mahmud G. Campbell C. J. Bishop K. J. M. Komarova Y. A. Chaga O. Soh S. (2009). Directing cell motions on micropatterned ratchets. Nat. Phys. 5, 606–612. 10.1038/nphys1306

[B79] Mandal K. Wang I. Vitiello E. Orellana L. A. C. Balland M. (2014). Cell dipole behaviour revealed by ECM sub-cellular geometry. Nat. Commun. 5:5749. 10.1038/ncomms6749 25494455

[B80] Misteli T. (2004). Spatial positioning; a new dimension in genome function. Cell. 119, 153–156. 10.1016/j.cell.2004.09.035 15479633

[B81] Mitrossilis D. Fouchard J. Pereira D. Postic F. Richert A. Saint-Jean M.. (2010). Real-time single-cell response to stiffness. Proc. Natl. Acad. Sci. U.S.A. 107, 16518–16523. 10.1073/pnas.1007940107 20823257 PMC2944728

[B82] Modol T. Brice N. Ruiz de Galarreta M. Garcia Garzon A. Iraburu M. J. Martínez–Irujo J. J.. (2014). Fibronectin peptides as potential regulators of hepatic fibrosis through apoptosis of hepatic stellate cells. J. Cell. Physiol. 230, 546–553. 10.1002/jcp.24714 24976518

[B83] Moeendarbary E. Harris A. R. (2014). Cell mechanics: principles, practices, and prospects. Wiley Interdiscip. Rev. Syst. Biol. Med. 6, 371–388. 10.1002/wsbm.1275 25269160 PMC4309479

[B84] Mohammed D. Charras G. Vercruysse E. Versaevel M. Lantoine J. Alaimo L. (2019). Substrate area confinement is a key determinant of cell velocity in collective migration. Nat. Phys. 10.1038/s41567-019-0543-3

[B85] Morse D. C. (1998). Viscoelasticity of concentrated isotropic solutions of semi-flexible polymers. 1. model and stress tensor; 2. linear response. Macromolecules 31, 7030–7044. 10.1021/ma9803032

[B86] Murthy S. E. Dubin A. E. Patapoutian A. (2017). Piezos thrive under pressure: mechanically activated ion channels in health and disease. Nat. Rev. Mol. Cell Biol. 18, 771–783. 10.1038/nrm.2017.92 28974772

[B87] Nagel G. Ollig D. Fuhrmann M. Kateriya S. Musti A. M. Bamberg E.. (2002). Channelrhodopsin-1: a light-gated proton channel in green algae. Science 296, 2395–2398. 10.1126/science.1072068 12089443

[B88] Nagel G. Szellas T. Huhn W. Kateriya S. Adeishvili N. Berthold P.. (2003). Channelrhodopsin-2, a directly light-gated cation-selective membrane channel. Proc. Natl. Acad. Sci. U.S.A. 100, 13940–13945. 10.1073/pnas.1936192100 14615590 PMC283525

[B89] Niland S. Cremer A. Fluck J. Eble J. A. Krieg T. Sollberg S.. (2001). Contraction-dependent apoptosis of normal dermal fibroblasts. J. Invest. Dermatol. 116, 686–692. 10.1046/j.1523-1747.2001.01342.x 11348456

[B90] Nourse J. L. Pathak M. M. (2017). How cells channel their stress: interplay between Piezo1 and the cytoskeleton. Semin. Cell Dev. Biol. 71, 3–12. 10.1016/j.semcdb.2017.06.018 28676421 PMC6070642

[B91] Parekh S. H. Chaudhuri O. Theriot J. A. Fletcher D. A. (2005). Loading history determines the velocity of actin-network growth. Nat. Cell Biol. 7, 1219–1223. 10.1038/ncb1336 16299496

[B92] Parker K. K. Brock A. L. Brangwynne C. Mannix R. J. Wang N. Ostuni E.. (2002). Directional control of lamellipodia extension by constraining cell shape and orienting cell tractional forces. FASEB J. 16, 1195–1204 10.1096/fj.02-0038com 12153987

[B93] Pathak A. Kumar S. (2012). Independent regulation of tumor cell migration by matrix stiffness and confinement. Proc. Natl. Acad. Sci. U.S.A. 109, 10334–10339. 10.1073/pnas.1118073109 22689955 PMC3387066

[B94] Peng A. W. Salles F. T. Pan B. Ricci A. J. (2011). Integrating the biophysical and molecular mechanisms of auditory hair cell mechanotransduction. Nat. Commun. 2:523. 10.1038/ncomms1533 22045002 PMC3418221

[B95] Plotnikov S. V. Pasapera A. M. Sabass B. Waterman C. M. (2012). Force fluctuations within focal adhesions mediate ECM-rigidity sensing to guide directed cell migration. Cell 151, 1513–1527. 10.1016/j.cell.2012.11.034 23260139 PMC3821979

[B96] Polio R. Rothenberg K. E. Stamenovic D. Smith M. L. (2012). A micropatterning and image processing approach to simplify measurement of cellular traction forces. Acta Biomater. 8, 82–88. 10.1016/j.actbio.2011.08.013 21884832 PMC3375107

[B97] Prass M. Jacobson K. Mogilner A. Radmacher M. (2006). Direct measurement of the lamellipodial protrusive force in a migrating cell. J. Cell Biol. 174, 767–772. 10.1083/jcb.200601159 16966418 PMC2064331

[B98] Raab M. Gentili M. de Belly H. Thiam H. R. Vargas P. Jimenez A. J.. (2016). ESCRT III repairs nuclear envelope ruptures during cell migration to limit DNA damage and cell death. Science 352, 359–362. 10.1126/science.aad7611 27013426

[B99] Riaz M. Versaevel M. Glinel K. Mohammed M. Gabriele S. (2016). Persistence of fan-shaped keratocytes is a matrix-rigidity-dependent mechanism that requires α5β1 integrin engagement. Sci. Rep. 6:34141. 10.1038/srep34141 27678055 PMC5039689

[B100] Roca-Cusachs P. Conte V. Trepat X. (2017). Quantifying forces in cell biology. Nat. Cell Biol. 19, 742–751. 10.1038/ncb3564 28628082

[B101] Roca-Cusachs P. del Rio A. Puklin-Faucher E. Gauthier N. C. Biais N. Sheetz M. P. (2013). Integrin-dependent force transmission to the extracellular matrix by α-actinin triggers adhesion maturation. Proc. Natl. Acad. Sci. U.S.A. 110, E1361–E1370. 10.1073/pnas.1220723110 23515331 PMC3625291

[B102] Roy B. Venkatachalapathy S. Ratna P. Wang Y. Jokhun D. S. Nagarajan M.. (2018). Laterally confined growth of cells induces nuclear reprogramming in the absence of exogenous biochemical factors. Proc. Natl. Acad. Sci. U.S.A. 115, E4741–E4750. 10.1073/pnas.1714770115 29735717 PMC6003522

[B103] Sabass B. Gardel M. L. Waterman C. M. Schwarz U. S. (2008). High resolution traction force microscopy based on experimental and computational advances. Biophys. J. 94, 207–220. 10.1529/biophysj.107.113670 17827246 PMC2134850

[B104] Sarkar R. Rybenkov V. V. (2016). A guide to magnetic tweezers and their application Front. Phys. 4:48 10.3389/fphy.2016.00048

[B105] Schwarz U. S. Soine J. R. (2015). Traction force microscopy on soft elastic substrates: a guide to recent computational advances. Biochim. Biophys. Acta 1853, 3095–3104. 10.1016/j.bbamcr.2015.05.028 26026889

[B106] Shao Y. Mann J. M. Chen W. Fu J. (2014). Global architecture of the F-actin cytoskeleton regulates cell shape-dependent endothelial mechanotransduction. Integr. Biol. 6, 300–311. 10.1039/c3ib40223a 24435061 PMC3963173

[B107] Shimamoto Y. Forth S. Kapoor T. M. Measuring (2015). Pushing and braking forces generated by ensembles of Kinesin-5 crosslinking two microtubules. Dev. Cell 34, 669–681. 10.1016/j.devcel.2015.08.017 26418296 PMC4604754

[B108] Shivashankar G. V. (2019). Mechanical regulation of genome architecture and cell-fate decision. Curr. Opin. Cell Biol. 56, 115–121. 10.1016/j.ceb.2018.12.001 30554028

[B109] Sleep J. Wilson D. Simmons R. Gratzer W. (1999). Elasticity of the red cell membrane and its relation to hemolytic disorders: an optical tweezers study. Biophys. J. 77, 3085–3095. 10.1016/S0006-3495(99)77139-0 10585930 PMC1300579

[B110] Solon J. Levental I. Sengupta K. Georges P. C. Janmey P. (2007). Fibroblast adaptation and stiffness matching to soft elastic substrates. Biophys. J. 93, 4453–4461. 10.1529/biophysj.106.101386 18045965 PMC2098710

[B111] Steinwachs J. Metzner C. Skodzek K. Lang N. Thievessen I. Mark C.. (2016). Three-dimensional force microscopy of cells in biopolymer networks. Nat. Methods 13, 171–176. 10.1038/nmeth.3685 26641311

[B112] Stephens A. D. Banigan E. J. Adam S. A. Goldman R. D. Marko J. F. (2017). Chromatin and lamin A determine two different mechanical response regimes of the cell nucleus. Mol. Biol. Cell. 28, 1984–1996. 10.1091/mbc.e16-09-0653 28057760 PMC5541848

[B113] Swift J. Discher D. E. (2014). The nuclear lamina is mechano-responsive to ECM elasticity in mature tissue. J. Cell Sci. 127, 3005–3015. 10.1242/jcs.149203 24963133 PMC4095853

[B114] Swift J. Ivanovska I. L. Buxboim A. Harada T. Dingal P. C. Pinter J.. (2013). Nuclear lamin-A scales with tissue stiffness and enhances matrix-directed differentiation. Science 341:1240104. 10.1126/science.1240104 23990565 PMC3976548

[B115] Tajik A. Zhang Y. Wei F. Sun J. Jia Q. Zhou W.. (2016). Transcription upregulation via force-induced direct stretching of chromatin. Nat. Mater. 15, 1287–1296. 10.1038/nmat4729 27548707 PMC5121013

[B116] Teixeira A. I. Abrams G. A. Bertics P. J. Murphy C. J. Nealey P. F. (2003). Epithelial contact guidance on well-defined micro- and nanostructured substrates. J. Cell Sci. 116, 1881–1892. 10.1242/jcs.00383 12692189 PMC1885893

[B117] Thery M. Racine V. Pepin A. Piel M. Chen Y. Sibarita J. B.. (2005). The extracellular matrix guides the orientation of the cell division axis. Nat. Cell Biol. 7, 947–953. 10.1038/ncb1307 16179950

[B118] Tomatsu I. Peng K. Kros A. (2011). Photoresponsive hydrogels for biomedical applications. Adv. Drug Delivery Rev. 63, 1257–1266. 10.1016/j.addr.2011.06.009 21745509

[B119] Trappman B. Gautrot J. E. Connelly J. T. Strange D. G. T. Yuan L. Oyen M. L. (2012). Extracellular-matrix tethering regulates stem-cell fate. Nat. Mater. 11, 642–649. 10.1038/nmat3339 22635042

[B120] Trepat X. Wasserman M. R. Angelini T. E. Millet E. Weitz D. A. Butler J. P. (2009). Physical forces during collective cell migration. Nat. Phys. 5, 426–430. 10.1038/nphys1269

[B121] Tsai M. A. Frank R. S. Waugh R. E. (1993). Passive mechanical behavior of human neutrophils: power-law fluid. Biophys. J. 65, 2078–2088. 10.1016/S0006-3495(93)81238-4 8298037 PMC1225943

[B122] Tseng Q. Wang I. Duchemin-Pelletier E. Azioune A. Carpi N. Gao J.. (2011). A new micropatterning method of soft substrates reveals that different tumorigenic signals can promote or reduce cell contraction levels. Lab Chip 11, 2231–2240. 10.1039/c0lc00641f 21523273

[B123] Uhler C. Shivashankar G. V. (2017). Regulation of genome organization and gene expression by nuclear mechanotransduction. Nat. Rev. Mol. Cell Biol. 18, 717–727. 10.1038/nrm.2017.101 29044247

[B124] Valon L. Marin-Llaurado W. T Charras G. Trepat X. (2017). Optogenetic control of cellular forces and mechanotransduction. Nat. Commun. 8:14396. 10.1038/ncomms14396 28186127 PMC5309899

[B125] Versaevel M. Braquenier J. B. Riaz M. Grevesse T. Lantoine J. Gabriele S. (2014a). Super-resolution microscopy reveals LINC complex recruitment at nuclear indentation sites. Sci. Rep. 4:7362. 10.1038/srep07362 25482017 PMC4258653

[B126] Versaevel M. Grevesse T. Gabriele S. (2012). Spatial coordination between cell and nuclear shape within micropatterned endothelial cells. Nat. Commun. 14:671 10.1038/ncomms1668 22334074

[B127] Versaevel M. Grevesse T. Riaz M. Lantoine J. Gabriele S. (2014b). Micropatterning Hydroxy-PAAm hydrogels and Sylgard 184 silicone elastomers with tunable elastic moduli. Methods Cell Biol. 121, 33–48. 10.1016/B978-0-12-800281-0.00003-8 24560501

[B128] Versaevel M. Riaz M. Corne T. Grevesse T. Lantoine J. Mohammed D.. (2017). Probing cytoskeletal pre-stress and nuclear mechanics in endothelial cells with spatiotemporally controlled (de-)adhesion kinetics on micropatterned substrates. Cell Adh. Migr. 11, 98–109. 10.1080/19336918.2016.1182290 27111836 PMC5308222

[B129] Versaevel M. Riaz M. Grevesse T. Gabriele S. (2013). Cell confinement: putting the squeeze on the nucleus. Soft Matter 9:6665 10.1039/c3sm00147d

[B130] Vining K. H. Stafford A. Mooney D. J. (2019). Sequential modes of crosslinking tune viscoelasticity of cell-instructive hydrogels. Biomaterials 188, 187–197. 10.1016/j.biomaterials.2018.10.013 30366219 PMC6279497

[B131] Vogel V. (2018). Unraveling the mechanobiology of extracellular matrix. Annu. Rev. Physiol. 80, 353–387. 10.1146/annurev-physiol-021317-121312 29433414

[B132] Wang Y. Nagarajan M. Uhler C. Shivashankar G. V. (2017). Orientation and repositioning of chromosomes correlate with cell geometry-dependent gene expression. Mol. Biol. Cell 28, 1997–2009. 10.1091/mbc.e16-12-0825 28615317 PMC5541849

[B133] Wang Y. L. Discher D. E. (2007). Cell mechanics, in Methods in Cell Biology, Vol. 83 (New York, NY: Elsevier), 521–524. 10.1016/S0091-679X(07)83026-317613323

[B134] Warmflash A. Sorre B. Etoc F. Siggia E. D. Brivanlou A. H. (2015). A method to recapitulate early embryonic spatial patterning in human embryonic stem cells. Nat. Methods 8, 847–854. 10.1038/nmeth.3016 PMC4341966 24973948

[B135] Whitesides G. M. Ostuni E. Takayama S. Jiang X. Ingber D. E. (2001). Soft lithography in biology and biochemistry. Annu. Rev. Biomed. Eng. 3, 335–373. 10.1146/annurev.bioeng.3.1.335 11447067

[B136] Wilson C. A. Tsuchida M. A. Allen G. M. Barnhart E. L. Applegate K. T. Yam P. T. (2010). Myosin II contributes to cell-scale actin network treadmilling through network disassembly. Nature 46, 373–379. 10.1038/nature08994 PMC3662466 20485438

[B137] Wirtz D. Konstantopoulos K. Searson P. C. (2011). The physics of cancer: the role of physical interactions and mechanical forces in metastasis. Nat. Rev. Cancer 11, 512–522. 10.1038/nrc3080. 21701513 PMC3262453

[B138] Xi W. Sonam S. Saw T. B. Ladoux B. Lim C. T. (2017). Emergent patterns of collective cell migration under tubular confinement. Nat. Commun. 8:1517. 10.1038/s41467-017-01390-x 29142242 PMC5688140

[B139] Xiaomeng L. Qingqing S. Qian L. Naoki K. Guoping C. (2018). Functional hydrogels with tunable structures and properties for tissue engineering applications. Front. Chem. 6:499 10.3389/fchem.2018.00499 30406081 PMC6204355

[B140] Xue X. Sun Y. Resto-Irizarry A. M. Yuan Y. Yong K. M. A. Zheng Y.. (2018). Mechanics-guided embryonic patterning of neuroectoderm tissue from human pluripotent stem cells. Nat. Mater. 17, 633–641. 10.1038/s41563-018-0082-9 29784997 PMC6622450

[B141] Yareni A. A. Pontes B. Ether D. S. Pires L. B. Araujo G. R. Frases S. (2016). Rheological properties of cells measured by optical tweezers BMC Biophys. 9:5 10.1186/s13628-016-0031-4 27340552 PMC4917937

[B142] Zemelman B. V. Nesnas N. Lee G. A. Miesenböck G. (2003). Photochemical gating of heterologous ion channels: remote control over genetically designated populations of neurons. Proc. Natl. Acad. Sci. U.S.A. 100, 1352–1357. 10.1073/pnas.242738899 12540832 PMC298776

[B143] Zhang X. Liu M. Li Y. Dong Y. Pingguan-Murphy B. Lu T. J. (2015). Engineering cell microenvironment using novel functional hydrogels. Eur. Polym. J. 72, 590–601. 10.1016/j.eurpolymj.2015.03.019

[B144] Zhang Y. Ge C. Zhu C. Salaita K. (2014). DNA-based digital tension probes reveal integrin forces during early cell adhesion. Nat. Commun. 5:5167. 10.1038/ncomms6167 25342432 PMC4209443

[B145] Zhao B. O'Brien C. Karunanayake Mudiyanselage A. P. K. K. Li N. Bagheri Y. Wu R.. (2017). Visualizing Intercellular Tensile Forces by DNA-based membrane molecular probes. J. Am. Chem. Soc. 139, 18182–18185. 10.1021/jacs.7b11176 29211468

[B146] Zhelev D. V. Needham D. Hochmuth R. M. (1994). Role of the membrane cortex in neutrophil deformation in small pipets. Biophys. J. 67, 696–705. 10.1016/S0006-3495(94)80529-6 7948682 PMC1225412

[B147] Zhou L. Cai M. Tong T. Wang H. (2017). Progress in the correlative atomic force microscopy and optical microscopy. Sensors 17:938. 10.3390/s17040938 28441775 PMC5426934

